# The APPLe Study: A Randomized, Community-Based, Placebo-Controlled Trial of Azithromycin for the Prevention of Preterm Birth, with Meta-Analysis

**DOI:** 10.1371/journal.pmed.1000191

**Published:** 2009-12-01

**Authors:** Nynke R. van den Broek, Sarah A. White, Mark Goodall, Chikondi Ntonya, Edith Kayira, George Kafulafula, James P. Neilson

**Affiliations:** 1 Liverpool School of Tropical Medicine, Liverpool, United Kingdom; 2 Malawi-Liverpool-Wellcome Trust Clinical Research Programme, Blantyre, Malawi; 3 Department of Obstetrics & Gynaecology, College of Medicine, University of Malawi, Blantyre, Malawi; 4 School of Reproductive & Developmental Medicine, University of Liverpool, Liverpool, United Kingdom; Cambridge University, United Kingdom

## Abstract

**Background:**

Premature birth is the major cause of perinatal mortality and morbidity in both high- and low-income countries. The causes of preterm labour are multiple but infection is important. We have previously described an unusually high incidence of preterm birth (20%) in an ultrasound-dated, rural, pregnant population in Southern Malawi with high burdens of infective morbidity. We have now studied the impact of routine prophylaxis with azithromycin as directly observed, single-dose therapy at two gestational windows to try to decrease the incidence of preterm birth.

**Methods and Findings:**

We randomized 2,297 pregnant women attending three rural and one peri-urban health centres in Southern Malawi to a placebo-controlled trial of oral azithromycin (1 g) given at 16–24 and 28–32 wk gestation. Gestational age was determined by ultrasound before 24 wk. Women and their infants were followed up until 6 wk post delivery. The primary outcome was incidence of preterm delivery, defined as <37 wk. Secondary outcomes were mean gestational age at delivery, perinatal mortality, birthweight, maternal malaria, and anaemia. Analysis was by intention to treat. There were no significant differences in outcome between the azithromycin group (*n* = 1,096) and the placebo group (*n* = 1,087) in respect of preterm birth (16.8% versus 17.4%), odds ratio (OR) 0.96, 95% confidence interval (0.76–1.21); mean gestational age at delivery (38.5 versus 38.4 weeks), mean difference 0.16 (−0.08 to 0.40); mean birthweight (3.03 versus 2.99 kg), mean difference 0.04 (−0.005 to 0.08); perinatal deaths (4.3% versus 5.0%), OR 0.85 (0.53–1.38); or maternal malarial parasitaemia (11.5% versus 10.1%), OR 1.11 (0.84–1.49) and anaemia (44.1% versus 41.3%) at 28–32 weeks, OR 1.07 (0.88–1.30). Meta-analysis of the primary outcome results with seven other studies of routine antibiotic prophylaxis in pregnancy (>6,200 pregnancies) shows no effect on preterm birth (relative risk 1.02, 95% confidence interval 0.86–1.22).

**Conclusions:**

This study provides no support for the use of antibiotics as routine prophylaxis to prevent preterm birth in high risk populations; prevention of preterm birth requires alternative strategies.

**Trial registration:**

Current Controlled Trials ISRCTN84023116

*Please see later in the article for the Editors' Summary*

## Abstract 2

In a randomized trial in Malawi of azithromycin versus placebo in over 2,000 pregnant women, Jim Neilson and colleagues show no benefit of azithromycin for a number of outcomes including preterm birth and prenatal death.

## Editors' Summary

**Background:**

Most pregnancies last about 40 weeks. Labor that occurs before 37 weeks of gestation (the period during which a baby develops in its mother) is defined as a preterm birth. In industrialized countries, 5%–10% of all births are preterm. Figures for preterm births are harder to obtain for low-income countries because of uncertainties about gestational dates but, in both rich and poor countries, preterm birth is a major cause of infant death and illness around the time of birth. Babies who are born prematurely also often have long-term health problems and disabilities. There are many reasons why some babies are born prematurely. Structural problems such as a weak cervix (the neck of the womb, which dilates during labor to allow the baby to leave the mother's body) can result in a premature delivery, as can pregnancy-induced diabetes, blood-clotting disorders, bacterial infections in the vagina or the womb, and malaria. However, it is impossible to predict which mothers will spontaneously deliver early.

**Why Was This Study Done?:**

At present there is no effective way to prevent premature births. Because infection is often associated with preterm labor and can occur early in pregnancy but remain undetected, one way to reduce the incidence of preterm births may be to give pregnant women antibiotics even when they have no obvious infection (prophylactic antibiotics). In this study, the researchers test this hypothesis by giving the antibiotic azithromycin to pregnant women living in Southern Malawi in a randomized, placebo-controlled trial. One baby in five is born before 37 weeks gestation in Southern Malawi and the women living in this part of sub-Saharan Africa have a high burden of infection. Azithromycin is a safe antibiotic that can treat many of the bacterial infections that have been implicated in preterm birth. It also has some antimalarial activity. In a randomized, placebo-controlled trial, participants are randomly assigned to receive a drug or identical-looking “dummy” tablets (placebo).

**What Did the Researchers Do and Find?:**

The researchers enrolled more than 2,000 pregnant women into the APPLe study (Azithromycin for the Prevention of Preterm Labor) and determined the gestational age of their unborn babies using ultrasound. Half of the women were given an oral dose of azithromycin at 16–24 weeks and at 28–32 weeks gestation. The remaining women were given a placebo at similar times. The mothers and their babies were followed up until 6 weeks after delivery. There was no significant difference in the primary outcome of the study—the incidence of delivery before 37 weeks gestation—between the two groups of women. Secondary outcomes—including mean gestational age at delivery, mean birth weight, and still births and infant deaths within a week of birth—were also similar in the two groups of women. Finally, the researchers did a meta-analysis (a statistical technique that combines the results of several studies) of their study and seven published studies of routine antibiotic prophylaxis in pregnancy, which indicated that the prophylactic use of antibiotics did not alter the risk of preterm birth.

**What Do These Findings Mean?:**

These findings provide no support for the use of antibiotics as prophylaxis to prevent preterm birth. The women included in this study had an unusually high incidence of preterm delivery and a high burden of infection so these findings may not be generalizable. The results of the meta-analysis, however, also provide no support for prophylactic antibiotics. Given that observational data have associated infection with preterm labor, why are the results of the APPLe trial and the meta-analysis negative? One possibility is that different antibiotics or dosing regimens might be more effective. Another possibility is that infection might be a secondary consequence of some other condition that causes preterm birth rather than the primary cause of early delivery. Whatever the reason for the lack of effect of prophylactic antibiotics, the researchers recommend that pregnant women should not be given antibiotics prophylactically to prevent preterm birth particularly since, in a recent study, the babies of women given antibiotics to halt ongoing preterm labor had an increased risk of developmental problems.

**Additional Information:**

Please access these Web sites via the online version of this summary at http://dx.doi.org/10.1371/journal.pmed.1000191.

- The March of Dimes, a nonprofit organization for pregnancy and baby health, provides information on preterm birth (in English and Spanish)
- The Nemours Foundation, another nonprofit organization for child health, also provides information on premature babies (in English and Spanish)
- Tommy's is a nonprofit organization that funds research and provides information on the causes and prevention of miscarriage, premature birth, and stillbirth
- The US Centers for Disease Control and Prevention provides information on maternal and infant health (in English and Spanish)
- The US National Women's Health Information Center has detailed information about pregnancy (in English and Spanish)
- MedlinePlus provides links to other information on premature babies (in English and Spanish)

## Introduction

Of the 4 million neonatal deaths each year, 99% occur in low-income countries and 28% are attributable to preterm birth [1]. Preterm delivery is one of the nine main causes of death in children below the age of 5 y [2]. Reducing the incidence of prematurity is important if Millennium Development Goal 4 for child survival (MDG-4) is to be achieved [2],[3] and important to reduce health service costs [4].

The incidence of preterm birth (before 37 completed wk of pregnancy) is between 5% and 10% in most industrialised countries [5]. A recently reported rise in preterm birth among primigravid women in Denmark from 3.8% to 5.7% [6] caused sufficient concern to merit an accompanying editorial [7]. The incidence of preterm birth is higher in the United States—rising from 10.7% in 1992 to 12.3% in 2003 [8]. Estimates in low-income countries are difficult because of common uncertainties about gestational age. However, we have previously reported much higher rates of 24% (95% confidence interval [CI] 21%–28%) and 20% (95% CI 17%–24%) in rural, community-based, ultrasound-dated studies in Malawi of, respectively, anaemic [9] and unselected [10] pregnant women. We are not aware of any other similar, rural studies from sub-Saharan Africa, although an urban study in Mozambique (using ultrasound) reported an incidence of 15% [11].

The causes of preterm labour are multiple, and the processes that ultimately lead to preterm birth may start many weeks before labour starts [12],[13]. There is compelling evidence for the etiological importance of infection, mainly ascending genital tract infection, and principally in association with earlier rather than later preterm birth [14],[15]. There is considerable evidence to suggest that intrauterine infection may occur quite early in pregnancy but remain undetected for months [14]. For example, women with high levels of C-reactive protein in early pregnancy have a much higher risk of spontaneous preterm birth (odds ratio [OR] 4.64, 95% CI 0.94–22.96) [16]. Thus, antibiotic prophylaxis to treat clinically unsuspected infection during pregnancy could, potentially, avoid later preterm births.

Our studied pregnant populations in Malawi carry high burdens of infective morbidity, including HIV (seropositivity 30%) [17], malaria (33%) [9], syphilis (10% positive *Treponema pallidum* haemagglutination [TPHA]), and other sexually transmitted infections, e.g., trichomoniasis 26%, candidiasis 37% (unpublished data). Anaemia is also common (haemoglobin <11 g/dl 72%) [18] and attributable not only to nutritional deficiencies but also to chronic inflammation. [19]

We hypothesised that routine antibiotic prophylaxis would decrease the incidence of preterm labour and birth, and conducted a placebo-controlled randomised trial of single-dose azithromycin 1 g orally at two time windows of pregnancy: 16–24 and 28–32 wk (Text S2). Azithromycin was chosen because of its broad spectrum of antibacterial activity including effectiveness against *Ureaplasma urealyticum* (implicated as an important cause of preterm labour), its efficacy against sexually transmitted infections including syphilis and chlamydia, its antimalarial effects (malaria is also a cause of prematurity), its safety profile in pregnancy [20], and the convenience of a single oral dose with few side-effects. A recently reported randomized trial showed that prophylactic azithromycin reduces the risk of miscarriage after amniocentesis [21].

We also hypothesised that routine azithromycin would decrease the incidence of malarial parasitaemia, because of its antimalarial properties [22],[23], and anaemia, because of the association of anaemia with chronic inflammation in this population [19].

At the time of planning our study, a Cochrane systematic review had been published on routine antibiotic administration to pregnant women; of six randomized trials, four reported preterm delivery rates (1,310 women) [24]. Pooled results from these diverse populations did not show a statistically significant reduction in the incidence of preterm delivery with prophylactic antibiotics (relative risk 0.88, 95% CI 0.71–1.08). but the wide CIs were compatible with a clinically important reduction in preterm birth.

Our aims were 2-fold. First, to investigate whether antibiotic prophylaxis would be of future practical benefit in the studied population in Malawi. Second, to test the intervention in the population with the highest reported incidence of preterm birth—as this could have generalizable importance to other high risk populations.

## Methods

### Participants and Setting

Women were recruited from three rural and one peri-urban antenatal clinic in Southern Malawi. Eligibility criteria were: gestational age less than 24 wk as determined by ultrasound (biparietal diameter measurement), intention to remain in the study area for the duration of the pregnancy, and signed informed consent. Biparietal diameter measurement [25] was performed by specially trained midwives and used to calculate gestational age (Concept 200l Dynamic Imaging). All women with confirmed gestational age <24 completed wk at this first visit were invited to participate in the trial.

Recruited women were randomly allocated to either 1 g azithromycin or placebo given at both 16–24 and 28–32 wk gestational windows. Antenatal care was provided to all women according to the usual schedule (planned 4-weekly visits until 32 wk; 2-weekly thereafter). At the booking visit, all women were screened for malaria (thick film), anaemia (Hb <11 g/dl by battery operated HemoCue device), and syphilis (VDRL). Haemoglobin and syphilis results were available on the same day; those found positive for syphilis were treated on the same day with intramuscular benzyl penicillin (1 g). All women received iron tablets daily (60 mg elemental iron as ferrous sulphate) with 0.25 mg folic acid, and antimalarial prophylaxis (two doses of Fansidar: 500 mg sulphadoxine with 25 mg pyrimethamine). All azithromycin (or placebo) and Fansidar tablets were taken under supervision at the clinic. Women who failed to attend for their 28–32 week visit were followed up, where possible, in the community.

Women were asked to report when they had delivered and to return for routine visits at 1 and 6 wk postnatally; women who withdrew from the study were followed up in an effort to obtain their delivery date and the survival status of the woman and her neonate.

### Outcome Measures

At booking and throughout antenatal care all women were encouraged to consider voluntary counselling and testing for HIV status, which was available in the clinic, as were antiretroviral drugs to prevent maternal to child transmission. We did not seek to collect prospective data about the HIV status of women. Our objective was to determine whether routine prophylactic treatment with an antibiotic in a population with a known high prevalence of infection and preterm labour would reduce the incidence of preterm labour (primary outcome). Secondary outcomes were mean gestational age at delivery, perinatal mortality, birthweight, and maternal malarial status and anaemia at 28–32 wk.

Preterm birth was defined as gestational age at delivery of at least 24 wk and less than 37 wk. Perinatal mortality included stillbirths and deaths within the first week of life.

We documented outcomes including date, type and place of delivery, type of assistance, and condition of mother and baby. For babies born in a hospital or health centre, birthweight was recorded. Babies were also weighed at postnatal visits at weeks 1 and 6.

### Sample Size

Ethical approval was obtained from the College of Medicine Research Ethics Committee (COMREC), Malawi, and permission to work at the Health Centres was obtained from the Ministry of Health in Malawi. The study was designed to have 90% power to detect a reduction in the incidence of preterm birth from 20% [10] to 15%, using a one-tailed test of significance at the 5% level. This required 987 women per arm. To account for an anticipated 15% dropout rate the total number recruited was to be 2,300. A one-tailed test was planned for the primary outcome since an increase in the incidence of preterm delivery would be of no more interest than equivalence [26],[27]. Two-tailed tests were planned for secondary outcomes, to ensure that an impact in either direction could be identified and reported. After agreeing to the analysis plan, a single interim analysis was performed using a significance level of 0.001 to avoid inflation of the final false positive error rate.

### Design

The randomization schedule was prepared by a statistician not involved in the trial analysis using a random generation procedure with variable block size to assign both treatments equally within each block of consecutive numbers. The azithromycin and placebo treatments allocated were provided as identical capsules (Pfizer) and packed in pairs of sealed envelopes for each individual study number, according to the randomization schedule, by staff who were not involved in the conduct of the trial. The randomization schedule was placed in sealed envelopes and not disclosed to anyone involved in the trial; it was only provided to the trial statistician for the interim and final analyses.

Numbers were assigned sequentially, by the study midwives, stratified by the two midwife teams, each serving two health centres, at the time of enrolment to the study. Both participants and study midwives were blinded to the study assignment. At no time during the study was there cause to unblind the treatment allocation for any participant.

### Analysis

In accordance with the analysis plan, logistic regression was used to estimate the effect of azithromycin on the incidence of preterm labour, prevalence of malaria parasitaemia at the 28–32-wk visit, and perinatal mortality. Analysis of covariance was used to estimate the effect of azithromycin on gestational age at delivery and on birth-weight. Variables included in these analyses as potentially influencing outcomes were: health centre, gravidity, body mass index (BMI), previous preterm delivery, anaemia, malaria, and syphilis status at the week 16–24 visit. Gestational age at delivery and multiplicity of pregnancy was also included in the analysis of birth-weight. Gestational age at delivery was also included (as linear and quadratic functions) in the analysis of perinatal mortality. All analyses were performed, using Stata software versions 9 or 10, on an intention-to-treat basis using all available data; for all secondary outcomes two tailed tests were performed using the 5% significance level.

An interim report, including analyses of safety and efficacy data for the 1,151 women with an estimated date of delivery prior to 8 February 2005 was prepared for the data and safety monitoring board in June 2005.

A limited meta-analysis was planned to include the results of this study together with the results of other randomized trials of routine antibiotic prophylaxis during pregnancy. These were identified using a comprehensive search of the Cochrane Pregnancy and Childbirth Database of Clinical Trials (details of search strategy not included). Only the primary outcome of the APPLe (Azithromycin for the Prevention of Preterm Labor) study (delivery <37 wk) was to be meta-analysed (Review Manager 5; Cochrane Collaboration). A random effects model was to be used if there was significant heterogeneity. There were no plans for subgroup or sensitivity analyses.

## Results

Over a period of 19 mo (February 2004 to September 2005) 11,713 women were seen for their first antenatal care visit in one of the four antenatal clinics. Of these 2,297 met the inclusion criteria and consented to enter the trial. Of the 9,416 women not recruited approximately 85% were more than 24 wk pregnant at this visit and 15% were either intending to move out of the area or did not want to join the study. The last follow-up visit was on 24 April 2006.

**Figure 1:**
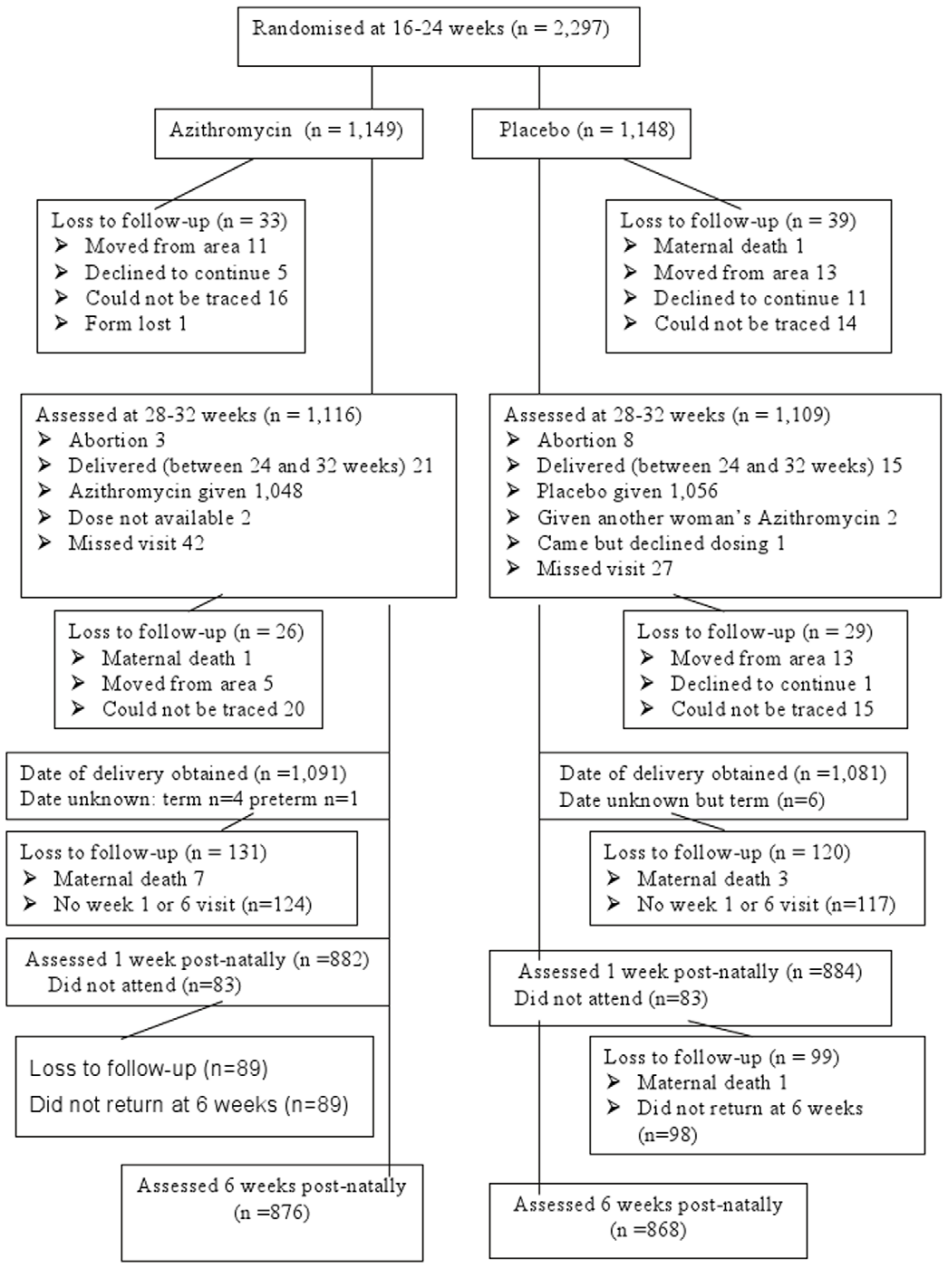
Trial profile.

A trial profile is presented in Figure 1. The primary outcome (whether delivery was preterm or not) was known for 2,183 (95.0%) women; 1,744 (75.9%) were followed up until 6 wk post partum. The following protocol deviations occurred (Text S1): (i) study numbers were assigned out of sequence on six occasions; (ii) three numbers were not assigned because of study drug shortage errors observed when opening envelopes; (iii) five women were recruited with gestational age >24 wk during the first 5 wk of recruitment (their gestational ages were all less than 25 wk by ultrasound scan) and five women were recruited at <6 wk. The second dose was received by 1,048 (91%) of women assigned to azithromycin and 1,056 (92%) of women assigned to placebo. 131 women received their second dose either before week 28 or after week 32; 14 (20) assigned to azithromycin (placebo) were early by up to 12 (30) d and 51 (46) were late by up to 20 (31) d. Two women (both randomised to placebo) received azithromycin in error (wrong envelope opened) at the second dose. The women for whom these doses were intended did not receive a second dose.

Baseline characteristics were similar for the two treatment groups (Table 1).

**Table 1:** Baseline comparability of randomised groups by treatment group.

Variable	Statistic/Category	Treatment Group	
		Azithromycin	Placebo
**Number of women**	—	1,149	1,148
**Gestational age at booking (wk)**	Mean (sd)	20.7 (2.1)	20.7(2.2)
**Maternal age (y)**	Mean (sd)	22.8 (5.1)	23.0 (5.2)
**Gravidity**	1	416 (36.2%)	397 (34.6%)
	2–4	581 (50.6%)	581 (50.6%)
	≥5	152 (13.2%)	170 (14.8%)
**Weight for height (kg/m^2^)**	Mean (sd)	22.7 (2.5)	22.7 (2.7)
**Syphilis status (VDRL + ve)**	—	81 (7.1%)	82 (7.1%)
**Haemoglobin (g/dl)**	Mean (sd)	10.7 (1.7)	10.8 (1.7)
**Positive malaria slide**	—	298 (25.9%)	274 (23.9%)

sd, standard deviation; VDRL, venereal disease research laboratory; + ve, positive.

The overall incidence of preterm birth was 17.1% and there was little difference between the treatment groups. The OR for preterm birth for women given azithromycin was 0.96 (one-sided 95% upper confidence limit: 1.21). Likewise, no statistically significant difference was found between the treatment arms for any of the secondary outcomes (Table 2). Although not prespecified as an outcome, there was also no statistically significant difference (Fisher's exact, *p* = 0.38) between the treatment arms in the incidence of early preterm birth (<34 wk): azithromycin (4.6%), placebo (5.4%).

**Table 2:** Summary and comparison of outcomes by treatment group.

Treatment Group	Treatment Group		*p*-Value*	Mean Difference or OR^a^	95% CI
	Azithromycin	Placebo			
**Number (%) who had preterm birth**	184/1,096 (16.8%)	189/1,087 (17.4%)	0.75 (0.71)	0.96^b^	(<1.21)^c^
**Mean gestational age (wk) at delivery**	38.5 (*n = *1,091)	38.4 (*n = *1,081)	0.18 (0.16)	0.16^d^	(−0.08 to 0.40)
**Mean birthweight (kg)**	3.03 (*n = *769)	2.99 (*n = *739)	0.08 (0.14)	0.04^d^	(−0.005 to 0.08)
***n*** ** (%) at 2nd dose with malaria parasitaemia**	117/1,014 (11.5%)	103/1,017 (10.1%)	0.46 (0.31)	1.11^b^	0.84–1.49
***n*** ** (%) at 2nd dose with anaemia** e	445/1,010 (44.1%)	418/1,017 (41.3%)	0.48 (0.24)	1.07^b^	0.88–1.30
***n*** ** (%) of perinatal deaths**	45/1,051 (4.3%)	51/1,035 (5.0%)	0.52 (0.48)	0.85^b^	(0.53–1.38)

Thirteen maternal deaths were reported; three occurred during pregnancy (one in the azithromycin group) and ten within 6 wk of delivery (seven in the azithromycin group). Adverse events were reported for three other women (vomiting after taking medication), of whom two were in the azithromycin group. The event rates for these deaths and adverse events were too low for statistical comparisons to be appropriate.

a Derived from multivariable analyses using women with available data.

b OR.

c One-sided 95% CI as specified in the analysis plan.

d Mean difference.

e This analysis was not specified in the analysis plan.

*p-Values for univariable analyses are given in parentheses.

**Figure 2:**
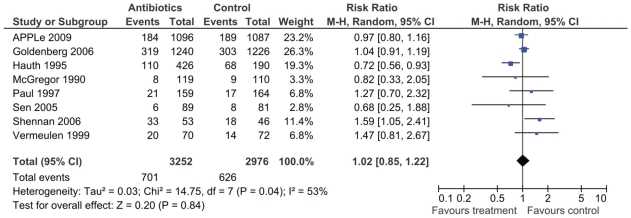
Random effects meta-analysis of trials of routine antibiotic prophylaxis in pregnancy that report preterm birth <37 wk as outcome.

Meta-analysis of the results of eight trials of routine antibiotic prophylaxis, including APPLe, using a random effects model, showed the relative risk of preterm birth (<37 wk) with routine prophylactic antibiotics to be 1.02 (95% CI 0.86–1.22) (Figure 2).

## Discussion

The overall incidence of preterm birth in our trial was 17.1%, which is higher than the figure reported in other populations, and which is not dissimilar to the findings of our previous, smaller study (incidence 20%; 95% CI 17%–24%) that formed the basis for the sample size calculation [10]. The incidence of preterm birth was the same for the two groups and our trial provided no support for our hypothesis that this regimen of prophylactic azithromycin would reduce the incidence of preterm birth and improve outcome.

Some researchers use early preterm birth (e.g., <34 wk) as their main outcome measure as neonatal mortality is higher after early preterm than late preterm birth. We chose, as the primary outcome, overall preterm birth (<37 wk) because our previous studies had shown high rates of perinatal mortality (160/1,000) associated with late preterm birth (33–36 wk) in this population [10]. In addition, morbidity is greater after late preterm than term birth, even in high income communities [28]. Azithromycin was, in any case, not shown in the current study to be effective in preventing early, as well as overall, preterm birth.

As far as we are aware, our studied population of unselected pregnant women in a rural population in sub-Saharan Africa is unique in having had the gestational ages of their pregnancies confirmed by ultrasound. Gestational dating by clinical examination in later pregnancy or by the date of the last menstrual period is unreliable. Many studies in low-income countries have therefore used “low birthweight” (<2.5 kg) as a surrogate for preterm birth—but it is a poor surrogate as low birthweight babies may be either small-for-gestational age at term or preterm. We are currently studying the mortality and morbidity and developmental outcome of these babies, with known gestational age at birth.

It has been convincingly argued that the results of clinical trials should be discussed against the background of the totality of evidence from other similar studies [29],[30]. Since the publication of the Cochrane review [24] that incorporated data from four studies [31]–[34], results from an additional four trials of routine antibiotic prophylaxis with preterm birth as an outcome have become available [35]–[37], including APPLe (Table 3). The largest trials, by far, are APPLe and HPTN 024. HPTN 024 was, like APPLe, performed in central Africa but relied, unlike APPLe, on menstrual dates and clinical examination rather than ultrasound for gestational age assessment [37],[38]. The eight trials took place in diverse settings (high and low income), with different types of participants (e.g., unselected women, women at high risk of preterm birth by past histories, women who were predominantly HIV positive), differing timings of treatment, and different antibiotic regimens. As well as clinical heterogeneity, there was statistical heterogeneity on analysis of the pooled data (I^2^, 51%) from, overall, 6,228 pregnancies. Meta-analysis, using a random effects model showed the relative risk of preterm birth (<37 wk) with routine prophylactic antibiotics to be 1.02 (95% CI 0.86–1.22).

**Table 3:** Randomised trials of antibiotic prophylaxis in pregnancy.

Study	Setting	Population	Gestation at Treatment (wk)	Treatment
**McGregor 1990 ** [**31**]	USA	235 unselected women	26–30	Erythromycin versus placebo
**Hauth 1995 ** [**32**]	USA	624 women at high risk of preterm birth	22–24	Metronidazole + erythromycin versus placebo
**Vermeulen 1995 ** [**33**]	Holland	168 women with history of preterm birth	26–32	Vaginal clindamycin versus placebo
**Paul 1997 ** [**34**]	India	437 unselected women	26–34	Erythromycin versus placebo
**Sen 2005 ** [**35**]	India	224 unselected “urban poor”	14–24	Metronidaxzole + cephalexin versus no treatment
**Shennan 2006 ** [**36**]	UK	100 high risk women with +ve fetal fibronectin	24–27	Metronidazole versus placebo
**Goldenberg 2006 ** [**37**],[**38**]	Zambia, Malawi, Tanzania	2,098 HIV+ and 335 HIV− women	20–24	Metronidazole + erythromycin versus placebo
**APPLe**	Malawi	2,297 unselected women	16–24 and 28–32	Azithromycin versus placebo

It is important to try to reconcile this finding that routine antibiotic prophylaxis does not prevent preterm birth, with the considerable observational data that associates infection with preterm labour. It is possible that different antibiotics or different antibiotic regimens with more intensive treatment schedules might impact on preterm birth rates. However, more complicated antibiotic regimens would have less appeal in resource-poor settings.

Another explanation is that ascending intrauterine infection may have been overemphasised as a primary cause of preterm birth. If factors such as psychosocial stress or heavy work, for example, are important in the premature triggering of the placental corticotropin-releasing hormone (CRH) pathway that ultimately leads to parturition [12], associated premature cervical shortening and dilatation might permit secondary ascending bacterial invasion of the uterine cavity. This has been suggested in the past [39] in the context of twin pregnancy in which preterm birth is common, and early cervical dilatation does occur [40]. Transvaginal ultrasound scanning has shown short cervices to be a powerful predictor of preterm birth in singleton pregnancies [41].

At the time of planning of the trial, it was assumed that antibiotic prophylaxis during pregnancy was unlikely to confer any harm, whether or not it conferred any benefit. The publication of the follow-up of the ORACLE trial has shown that this assumption was wrong. This report showed that children of women treated with antibiotics for preterm labour (not prophylactically) were more likely to have neuro-developmental delay [42]. Our study adds further weight to the conclusion that pregnant women should not be treated with antibiotics unless for specific infections and with good evidence of likely benefit.

## Supporting Information

Text S1 Trial protocol.
(0.07 MB DOC)

Text S2 CONSORT checklist.
(0.06 MB DOC)

## Abbreviations

CI: confidence interval
OR: odds ratio
