# Supplementary material for: The APPLe Study: A Randomized, Community-Based, Placebo-Controlled Trial of Azithromycin for the Prevention of Preterm Birth, with Meta-Analysis
Source: PLoS Med. 2009 Dec 1;6(12):e1000191. doi: 10.1371/journal.pmed.1000191 (PMC2776277; doi:10.1371/journal.pmed.1000191)
Supplement: Text S1 — Trial protocol. (0.07 MB DOC) [file pmed.1000191.s001.doc]

Title:

Prevention of infection associated premature delivery in a rural population in Malawi.

**Investigators:**

**Dr N van den Broek**

**Mrs B Kambala**

## Co-investigators/ Advisers

**Prof EN Tadesse**

**Dr SA White**

**Prof ME Molyneux**

**Prof J Neilson**

**Institutions involved:**

**College of Medicine Malawi (Department of Obstetrics and Gynaecology)**

**Malawi-Liverpool-Wellcome Trust Clinical Research Programme**

**University of Liverpool**

**Executive Summary:**

Premature delivery makes a large contribution to perinatal and infant mortality and disability. Recurrent pregnancy loss through prematurity may also produce significant risk to the mother in resource-poor areas where the risks of maternal mortality and morbidity are high. We have previously found a very high risk of premature labour and delivery (20-25%) in a rural area of Malawi, Namitambo. Uniquely this was a rural community based study in which all women had gestational age established with precision by ultrasound examination in early to mid pregnancy. It is well established that infection may cause premature labour, and we found a high prevalence of vaginal infection in this community as well as biochemical evidence of chronic infection. Previous work in Kenya (in urban populations that did not have gestational age confirmed by ultrasound) has suggested that antibiotic prophylaxis could improve outcome of pregnancy in populations with high burdens of infective disease. We propose therefore to perform a randomised, placebo-controlled trial of an antibiotic intervention at 16-20 and 28-32 weeks of pregnancy. The primary outcome measures in the trial will be the incidence of preterm birth and the mean gestational age at delivery. We will at the same time study the possible impact of antibiotic prophylaxis on maternal anaemia – as previous studies have suggested that chronic infection may be important in the aetiology of anaemia in pregnancy. We will also explore views among women (and men) on premature labour and fetal and neonatal loss.

# Introduction and Rationale:

In developing countries perinatal mortality continues to be unacceptably high with estimates for sub-Saharan Africa ranging between 45 and 75 per 1000 ^1^, compared to 8 per 1000 for the UK. The major cause of perinatal death world-wide is pre-mature (pre-term) delivery (i.e. at < 37 weeks gestation). The incidence of premature delivery in most industrialised countries is reported to be around 7%, but there are few reliable data from sub-Saharan Africa. We have recently completed a Wellcome Trust supported randomised trial of Vitamin A supplementation in pregnant, anaemic women in a rural community in Malawi (Namitambo) and, unexpectedly found a very high incidence of premature labour among participants. Gestational age was determined by ultrasonography before 24 weeks gestation. Mean gestational age at booking (= first antenatal clinic visit) was 20.6 weeks (s.d 3.5). Information about delivery was available for 589 women. For women who were non-anaemic at booking premature delivery was recorded in 20.7% of cases, for anaemic women ([Hb] < 11.0 g/dl) premature delivery was recorded in 24.7% of cases and for women with sever anaemia ( [Hb] < 8.0 g/dl) prematurity was recorded in 29.7% of cases. ^2, 2b^ All of these women had, by virtue of entry criteria to the study, singleton pregnancies (multiple pregnancy carries a particularly high risk of preterm delivery). They also received oral iron and folic acid supplementation, and two routine doses of anti-malarial prophylaxis (SP) in the antenatal clinic. Importantly this was a population which had undergone mid-pregnancy ultrasound dating as part of the research protocol, so the gestational age at delivery was not in doubt. Many studies have used low birth weight (i.e. <2.5 kg) as a surrogate measure of prematurity but this index cannot distinguish between preterm birth and fetal growth restriction. We are aware of only one other such study in Africa with ultrasound dating; this was done in an urban centre (Maputo, Mozambique) and reported a 15% incidence of preterm delivery with low maternal weight gain and malaria as major risk factors ^3^. Another study in rural Malawi (Mangochi) ^4^ described a 22% incidence of premature delivery in an unselected population, but gestational age assessment was based on tape measure of symphysis-fundal height (uterine size) - a method recognised as inaccurate in determining the duration of pregnancies.

The consequences of a high incidence of preterm delivery are considerable because of the contribution to neonatal mortality – 30% in one rural area of the Gambia ^5^ and 65% in the Mangochi study in Malawi^4^. In a series from Tanzania, 52% of all babies with respiratory distress syndrome (closely associated with prematurity) died^6^. Prematurity also makes an important contribution to later, infant mortality, at least as significant a contribution as maternal HIV infection in one recent report from Malawi^7^. Where the facilities exist in hospitals for neonatal care, prematurity represents the major indication for special care, with significant health cost consequences. ^8,9^

Prematurity is also well recognised as conferring long-term neuro- developmental disadvantages that include, at one end of the spectrum, cerebral palsy, and at the other more subtle educational and social difficulties. It is clearly undesirable for populations already disadvantaged by poverty and high burdens of disease, to also cope with the short and long term consequences of preterm birth.

There may be an additional dimension to the problem that has not, to our knowledge been highlighted before. Women who have had one premature delivery are at increased risk of this complication recurring in future pregnancies. It is now well recognised from epidemiological work that the dangers associated with high parity are in part explained by the efforts of women at reproductive disadvantage to achieve their desired family size. It is important to investigate this possibility in Namitambo because of the much higher risk of maternal death and morbidity per pregnancy in rural Malawi – we would like to explore this further and will obtain both women’s and men’s views on these issues.

Since our population in Namitambo were all anaemic for entry into the Vitamin A trial, the possibility has to be considered that the anaemia per se was responsible for the high rates of premature delivery (we have found 72% of this population to be anaemic during pregnancy as defined by a haemoglobin < 11 g/dl)^10^. In fact the link between anaemia itself and preterm delivery is, at best, weak. A large UK study found that the incidence of preterm delivery was 9.7% in women with haemoglobin levels < 8.5 g/dl compared with the national average of 7% and incidences at high haemoglobin levels of 13.5-14.5 g/dl and >14.5 g/dl were respectively, 12% and 22.5% ^11^. We hypothesise that any association between anaemia and premature delivery in the Namitambo population is indirect and explained by a common link of chronic infection. We have also found, in Namitambo, that 20% of anaemic women had no demonstrable deficiencies of iron, Vitamin A, folate or Vitamin B12, but more than half of these women had high C reactive protein (CRP) levels, a marker of infection ^12^. Similarly in 73% of iron-replete but anaemic women, there were high levels of CRP, suggesting that chronic infection might be responsible for physiological failure to mobilise iron stores. In the Vitamin A trial, we found 74% of women to have CRP levels > 5mg/dl and 23% to have levels > 20 mg/dl. In HIV +ve women in Namitambo we found anaemia to be more common than in HIV –ve women, but could find no nutritional basis for the differences. ^13^

There are compelling data to link infection, and especially, lower genital tract infection with preterm labour and delivery.^14-16^. We have recently completed a survey of genital tract infection in the pregnant population of Namitambo and this is currently being analysed. 1000 women were screened by venepuncture and vaginal speculum examination for high vaginal and endocervical swabs for microbiological studies. Currently available results have been obtained from the first 565 and showed 10% to be VDRL (+ve) (syphilis), 21% to be HIV positive, 3% to have asymptomatic urinary tract infection, 78% to have abnormal vaginal microbiology, including candidiasis 40%, trichomonas 25% and clue cells (bacterial vaginosis) in 17%. We were unable to test for gonorrhoea but found pus cells in 15% of women screened. Chlamydial testing will be completed in the laboratory shortly. This is thus a population with a high prevalence of abnormal vaginal flora. (unpublished information). Importantly there was no relationship between reported symptoms and/or findings on speculum examination and type or severity of infection. It has been known for some time now that the ‘syndromic approach’ to treatment of sexually transmitted infections in women is poor in accuracy. This may be particularly so in pregnancy when vaginal discharge is also noted to be different as a result of hormonal changes.

The biochemical and cellular events that occur at the time of labour resemble an inflammatory response with, in particular, cytokine release (notably IL-6 and TNF) ^17^. Women in labour have raised serum IL-6 concentrations; in women whose labour is premature labour and is associated with intrauterine infection, IL-6 is extremely elevated. ^18^ IL-6 stimulates the production of Prostaglandin E2 that in turn stimulates uterine contractions and cervical ripening. Similarly TNFa is elevated in preterm labour, and very elevated if this is associated with microbial invasion of the amniotic cavity ^19^.

There are only two trials of untargeted antibiotic prophylaxis (i.e. not targeted at specific cultured micro-organisms) in African pregnant populations with high prevalence of lower genital tract infections including sexually transmitted infections. Both studies were performed in urban populations in Nairobi, Kenya, by the same research team. In the first study ^20^, 400 women were randomly allocated to receive a single dose intramuscular injection of a cephalosporin antibiotic at an estimated 28-32 weeks of pregnancy or placebo. Mean birth weight was greater in the antibiotic arm (3209 g vs 3056 g) and, though there were fewer low birth weight babies (4.0 % vs 9.3%) this difference did not reach conventional levels of statistical significance. The second trial ^21^ specifically addressed high risk pregnant women; 320 women with poor obstetric histories (including histories of previous preterm deliveries) were randomly allocated to receive the same antibiotic treatment as the first study, or placebo. There were statistically significant increases in mean birth weight (2927g vs 2772g) and decreases in the number of low birth weight babies (18.7% vs 32.8%) among women in the antibiotic treated arm. Mean gestational age at delivery was not statistically, significantly different but in both trials most women were offered treatment for the first time late in pregnancy (third trimester) and the authors acknowledge the uncertainties of gestational age estimation in their study populations.

These findings are sufficiently encouraging to support a further trial in a similar population with high levels of infection but with gestational age firmly established by mid-pregnancy ultrasound examination- the Namitambo-Chiradzulu field site would provide the ideal setting. If the Kenyan findings are confirmed, this would provide important information for health promotion in lager areas of Africa and elsewhere.

Antenatal coverage in the Namitambo-Chiradzulu area is excellent with 95% of women attending antenatal and postnatal clinics. An average of 5.2 (sd 2.6) clinic visits is made during pregnancy.^22^ Most women (63%) deliver at the health centre or district hospital. In 19% of cases assistance at delivery is by an untrained traditional birth attendant at home and in 16% of cases assistance at delivery is by a female relative. The rest of the deliveries are unattended.^22^ Women who deliver at home are usually seen at nearest health centre in the first week post delivery as part of postnatal care.

Perinatal mortality is estimated at 30 per 1000 births, and maternal mortality at 413 per 100,000 deliveries.^22^ Women between the ages of 15 and 45 were interviewed and asked about pregnancy outcome; 8.9% of women reported the loss of at least one pregnancy before 7 months, 8% had lost a pregnancy after 7 months and 4.3% of women reported the loss of a baby within the first 24 hours of birth. Gestational age is not defined in weeks in this population but culturally a distinction is made between loss before or after ‘7 months’ of pregnancy.

# Objectives of the study

- To confirm the high incidence of premature labour and delivery in an unselected, rural pregnant population in southern Malawi (Namitambo-Chiradzulu) in whom gestational ages had been determined by ultrasound.
- To determine the effects of prophylactic treatment with antibiotic given at 16-22 and 28-32 weeks of pregnancy on premature delivery, gestational age at delivery, perinatal mortality, maternal anaemia, and other pregnancy outcomes, by a randomised controlled trial.
- To explore knowledge of, and attitudes to, premature labour and fetal and neonatal loss among women and men in the Namitambo area.

# Methods

We propose a randomised placebo-controlled trial to assess the significance of infection as a cause of prematurity, and to test a possibly effective intervention strategy in this high-risk population. Women will be eligible for entry into the study if they have a single viable pregnancy with fetal measurement (biparietal diameter) < the mean for 24 weeks gestation - gestational age is accurate to within 10 days at this phase of pregnancy. Standard obstetric epidemiological data, including those denoting increased risk of premature delivery will be noted.

Women will be randomly allocated by opening a sealed, numbered pre-prepared envelope to one of the two interventions. In the experimental arm they will receive antibiotic treatment on a single occasion between 16-24 weeks (at the time of booking) and again as a second single dose between 28 and 32 weeks (the time of the intervention in the Kenyan studies). Women in the control group will receive oral placebo on both occasions.

For the antibiotic, we propose to use **Azithromycin** **1 gram** given as a single oral dose. Azithromycin is chosen because of its wide spectrum of activity against micro-organisms likely to be associated with preterm labour.^23^ It also has the advantage of single dose administration, and will likely become less expensive in the near future as the patent expires.

Azithromycin is safe in pregnancy. It is a macrolide antibiotic which is active against a wide range of organisms which are associated with premature labour including, *Neisseria gonorrhoeae*, *Escheridia coli,* *Salmonella* spp., *Chlamydia* and bacterial vaginosis. In addition it is active against gram-negative organisms that have been associated with adverse pregnancy outcome including B haemolytic- streptococci and *Bacteroides spp*.^23^ Azithromycin is similar to erythromycin but has greater activity than erythromycin against gram-negative bacteria and improved pharmacokinetics with the added advantage that it can be given as a single dose. Mild gastro-intestinal side effects have been noted but otherwise Azithromycin is very well tolerated.^23^ The gastro-intestinal side effects were noted in one study where 2g doses were given and vomiting was reported in up to 8% of patients. We will give a 1g dose under direct supervision and ask women to report any side effects noted.

There is some very recent evidence that azithromycin may also have a protective effect against malaria (personal communication, Pfizer April 2002). We will therefore also monitor malaria parasitaemia during pregnancy.

In addition to antibiotic treatment or placebo all women will receive daily supplements of iron (with folic acid) as well as the recommended two doses of SP as presumptive treatment for malaria. Antenatal and postnatal care will be provided in accordance with current Ministry of Health and Population (MOHP) guidelines. Vitamin A 200 000 IU will be provided for postnatal women as is currently recommended by the MOHP. In accordance with international recommendation women in the postnatal period will also be offered iron supplementation.

Women will be recruited at antenatal clinics in the Chiradzulu district. Clinics, in which we currently work or have worked, include the Namitambo Health Centre, Chiradzulu Hospital Nguludi Hospital and Mikolongwe clinic. Each clinic books between 50-100 new antenatal clinic attendees per month. We are aware from previous work that there is widespread acceptance within this area of recruitment into research studies both by patients and by staff working in these clinics. From previous experience we would anticipate recruitment rates of at least 150 women per month.

All women are eligible to enter the trial if they so wish if they are less than 24 weeks pregnant by ultrasound scan measurement.

As part of this trial all women who book for antenatal care at the site will be offered a haemoglobin test, malaria test and screening for syphilis (VDRL) at booking. This will also include women who decide not to enter the trial. Azithromycin is not currently recommended for the treatment of syphilis. Women who test positive for VDRL will be offered benzyl penicillin i.m. according to current Malawi Ministry of Health recommendation. We will endeavour to provide testing on site so that women can be offered treatment the same day. Partners of women who test positive for syphilis will be encouraged to come to the health facility for treatment. A second (venous) blood sample will be taken at 30-32 weeks and tests for malaria, haemoglobin and a VDRL will be repeated.

At other follow-up visits screening for anaemia and malaria will be offered. This will involve a finger prick sample of blood being taken only. In the postnatal period (week 1 postnatal or if not attending week 6) women will again be offered screening.

An Ultrasound Scan to estimate gestational age will be offered at booking and repeated at 30-32 weeks. Ultrasonography will also be available for other obstetric indications e.g. failure to feel movement, suspected intrauterine growth retardation, suspected placenta praevia etc. this will be available for all women attending the clinics i.e. not exclusively those who decide to enter the trial. Midwives attending patients will receive extra training so that they are competent to perform these scans.

Women will be encouraged to undergo screening for HIV, with appropriate pre- and post-test counselling, and **nevirapine** will be made available for patients who are HIV positive and who wish to receive nevirapine to reduce mother to child transmission. MSF are currently in process of setting this up in both Nguludi and Chiradzulu Hospitals and we will work together with them. HIV testing will however not be a pre-requisite for recruitment into this trial.

For the study of women and men’s opinion on premature delivery and pregnancy loss, focus group discussions will be organised. We hope to solicit the help of social scientists from Malawi and the Liverpool School of Tropical Medicine for this aspect of the work.

The study period is anticipated to be 3 to 4 years.

### Sample size

With an estimated incidence of 20% of premature labour, in order to detect a reduction from 20% to 15% with 90% power and a 1 tailed test of significance at the 5% level, 987 women are needed per arm. With a 15% dropout rate this would mean a total of 2 270 women recruited into this trial. If the incidence of premature labour should prove to be 15% this sample size will detect a reduction of 4.6% from 15% to 10.4% (with 90% power).

### Data collection

Data collection will be using both the standard antenatal cards as used by the Ministry of Health and specific forms designed for the trial (copies enclosed). The antenatal cards are patient-carried cards. All laboratory results will be entered on antenatal cards as well as trial forms.

### Data management and analysis

An independent data safety and monitoring committee (DSMC) will be established to monitor this trial if the trial is approved. Data will be double-entered and verified in Microsoft Access. An interim analysis is planned for which the statistician on the DSMC will be provided with the database. Primary outcome measure is incidence of premature labour (using gestational age as determined by Ultrasound scan at booking and date of delivery). Analysis of the primary outcome will be using a two by two table and Pearson X^2^ statistic. Secondary outcomes include mean gestational age, perinatal mortality and birthweight. Association with anaemia status, iron status and malaria parisitaemia will be examined using logistic regression.

### **Presentation and dissemination of results**

Results of this trial will be presented at both local and international meetings. Results will first be presented and discussed in local meetings such as the College of Medicine’s annual Research Dissemination Meeting, weekly departmental meetings in the Dept of Obstetrics & Gynaecology, COM and QECH, and the Wellcome Trust annual meeting. Results will also be disseminated to the community in which work has taken place and to staff from the various sites involved in the project.

Publication of results will be in the Malawi Medical Journal and in regional and international journals.

# Ethical considerations

Consent forms are enclosed with this application. The trial will be introduced to the community via a series of community meetings. This has been successfully organised in the past via the relevant local community leaders. The trial will also be explained at antenatal clinics both as part of information given to the group of women attending as a whole as well as to individual women who are attended to.

As has been our practice in the past, the team of midwives and laboratory technicians from the project will work together with existing staff in the institutions in which we work such that all staff together provide optimum ante- and post-natal care. Services such as blood testing for haemoglobin, malaria parasitaemia, screening for syphilis and ultrasonography will be available to all women attending the clinics i.e. not only to women who agree to enter the proposed trial.

**Possible constraints**

Problems with recruitment:

This is a large trial and we may have problems recruiting sufficient numbers of women into the trial. We will establish two teams of midwives who will work at up to four selected sites together with existing staff. As a result of previous work in the district we have established good links both with the communities and staff of the health facilities in this area. Should recruitment be insufficient from the sites chosen we will expand to recruit in two further sites such as Bangwe Health Centre and/or PIM clinic.

Loss to follow up:

We are aware from previous work that it may not be possible to follow up all patients throughout their pregnancies and into the postnatal period. This may be because people decide to discontinue with the trial e.g. because they are not happy to provide a (further) blood sample, because they move or because they lose the index pregnancy e.g. a late miscarriage. All efforts will be made to visit women who are no longer attending clinic at home. Women who wish to discontinue with the trial will continue to receive antenatal and postnatal care provided by the team. From previous experience we estimate a dropout rate of 15% and have adjusted the sample size accordingly.

##### Training

Training will be organised for staff working at the various facilities, which will focus on premature delivery, infections and malaria. A series of ten 3-day workshops is envisaged

The Wellcome Trust will be approached to fund staff to attend the 3-month course in Reproductive Health for Developing Countries. This is run jointly by the Royal College of Obstetricians (RCOG) and the Liverpool School of Tropical Medicine. This course leads to a Diploma in Reproductive Health from the RCOG.

It is anticipated that at least 2 people from the Department of Obstetrics and Gynaecology will be selected by the Department of Obstetrics and Gynaecology to obtain this Diploma. In addition funding will actively be sought from the Liverpool School of Tropical Medicine, The British Council and the Dutch Government to enable more people to attend this course.

**References.**

1. Greenwood AM, Greenwood BM, Bradley AK, Williams K, Shenton FC, Tullock S, Byast P, Oldfield FSJ. A prospective survey of the outcome of pregnancy in a rural area of the Gambia. Bulletin of the WHO 1987:65:635-643
2. van den Broek NR, White SA, Mhango C, Cook JD, Letsky EA, Molyneux M, Neilson JP. Double blind randomised trial of antenatal Vitamin A supplementation in pregnant anaemic women in rural Malawi (in preparation)

**2b. Ntonya C, Maganga J, Kambala B, Mhango E, Mwafulirwa W, van den Broek NR.**

**The incidence of premature labour. Malawi Research Dissemination Day 2001.**

1. Osman NB, Challis K,Cotiro M, Nordahl G, Bergstrom S. Perinatal outcome in an obstetric cohort of Mozambican women. Journal of Tropical Paediatrics 2001;47:30-38
2. Kulmala T, Vaahtera M, Ndekha M, Koivisto AM, Cullinan T, Salin ML, Ashorn P. The importance of preterm births for peri- and neonatal mortality in rural Malawi. Paed Perinatal Epidemiology 2000;14:219-226
3. Leach A, McArdle TF, Banya WA, Krubally O, Greenwood AM, Rands C, Adegbola R, de Francisco A, Greenwood BM. Neonatal mortality in a rural area of The Gambia. Ann Trop Paed 1999;19:33-43
4. Mlay GS, Manji KP. Respiratory distress syndrome among neonates admitted at Muhimbili Medical Centre, Dar es Salaam, Tanzania. J Trop Paeds 2000;46:303-307
5. Vaahtera M. Kulmala T, Ndekha M, Koivisto AM, Cullinan T, Salin ML, Ashorn P. Antenatal and perinatal predictors of infant mortality in rural Malawi. Arch Dis Child 2000;82:S200-204
6. Kambarami RA, Matibe P, Pirie D. Risk factors for neonatal mortality: Harare Central Hospital Neonatal Unit, Zimbabwe. Centr Afr J Med 1999;45:169-173
7. Wilkinson D, Connoly C, Stirling S. Impact of prematurity on admissions to the neonatal nursery of a rural South African district hospital. J Trop Paeds 1999;45:76-80
8. **van den Broek NR, Rogerson SJ, Mhango CG, Kambala B, White SA, Molyneux ME. Anaemia in pregnancy in southern Malawi: prevalence and risk factors. BJOG 2000:107;445-451**
9. Steer P, Alam MA, Wadsworth J, Welch A. Relation between maternal haemoglobin concentration and birth weight in different ethnic groups. BMJ 1995;310:489-491
10. **van den Broek NR, Letsky EA, Etiology of anaemia in pregnancy in south Malawi. Am J Clin Nutr 2000;72:247S-256**
11. **van den Broek NR, White SA, Neilson JP. The relationship between asymptomatic HIV infection and the prevalence and severity of anaemia in pregnant Malawian women. Am J Trop Med Hyg 1998;59:1004-1007**
12. Romero R,Mazor M, Wu YK. Infection and the pathogenesis of preterm labour. Seminars in Perinatology 1988;12:262-279
13. Lamont RF, Fisk N. The role of infection in the pathogenesis of preterm labour. Progress in Obstetrica dn Gynaecology 1993:10:135-158
14. Sebire NJ. Choriodecidual inflammatory syndrome (CoDIS) is the leading and underrecognised cause of preterm delivery and second trimester miscarriage. Med Hypothesis 2001;56:497-500
15. Keelan JA, Coleman M, Mitchell MD. The molecular mechanisms of term and preterm labour: recent progress and clinical implications. Clin Obstetrics and Gynecology 1997;90:465-469
16. Greig PC, Murtha AP, Jimmerson CE et al. Maternal serum interleukin 6 during pregnancy and during preterm labour. Am J Obst Gynecol 1997;90:465-469
17. Maymon E, Ghezzi F, Edwin SS, Mazor M, Yoon BH,Gomez R,Romero R. The tumor necrosis factor alpha and its soluble receptor profile in term and preterm parturition. Am J Obstet Gynecol 1999;181:1142-1148
18. Temmerman M, Njagi E, Nagelkerke N, Ndinya-Achola J, Plummer FA, Meheus A. Mass antimicrobial treatment in pregnancy. A randomised-placebo-controlled trial in a population wit high rates of sexually transmitted diseases. J Reprod Med 1995;40:176-80
19. Gichangi PB,Ndinya- Achola JO, Ombete J, Nagelkerke NJ, Temmerman M. Antimicrobial prophylaxis in pregnancy: a randomised placebo-controlled trial with cefetamet-pivoxil in pregnant women with a poor obstetric history. Am J Obstet Gynecol 1997;177:680-684
20. **van den Broek NR, White SA, Ntonya C, Ngwale M, Cullinan T, Molyneux ME, Neilson JP. A population based reproductive health survey from south Malawi- Effect of education, distance from Health Centre and head of household on reproductive health seeking behaviour and outcomes. (submitted)**
21. Kucers A, Crowe SM, Graysin ML,Hoy JF. The Use of antibiotics. A clinical review of antibacterial,antifungal and antiviral drugs. Azithromycin. Publishers: Butterworth and Heinemann. 1997: 653-662.

**NB copies of papers in bold have been submitted previously to the COMRC and can be provided again if required.**
